# Exercise Science Graduates in the Healthcare System: A Comparison Between Australia and Switzerland

**DOI:** 10.3389/fspor.2022.766641

**Published:** 2022-03-28

**Authors:** Justin Carrard, Maurin Gut, Ilaria Croci, Stephen McMahon, Boris Gojanovic, Timo Hinrichs, Arno Schmidt-Trucksäss

**Affiliations:** ^1^Division of Sports and Exercise Medicine, Department of Sport, Exercise and Health, University of Basel, Basel, Switzerland; ^2^K.G. Jebsen Center of Exercise in Medicine, Department of Circulation and Medical Imaging, Faculty of Medicine, Norwegian University of Science and Technology, Trondheim, Norway; ^3^Emergency Department and Orthopaedics Unit, Ballarat Health Services, Ballarat, VIC, Australia; ^4^Sports Medicine, Swiss Olympic Medical Center, Hôpital de la Tour, Meyrin, Switzerland; ^5^Centre SportAdo, Woman-Mother-Child Department (DFME), Lausanne University and Hospital (CHUV), Lausanne, Switzerland

**Keywords:** sport scientists, exercise therapists, sport physiologists, healthcare, active living, non-communicable disease (NCD)

## Abstract

Physical inactivity (PI) is a leading risk factor for global mortality worldwide, a major preventable cause of non-communicable diseases (NCDs) and a socioeconomic burden for healthcare systems. Fortunately, evidence shows that exercise interventions delivered by qualified exercise science graduates is an effective way to reduce PI, prevent and treat NCDs. This study compares the integration of exercise science graduates, defined as university graduates with degrees in sport and exercise science, in the healthcare systems of Australia, a commonly cited model in this regard, and Switzerland, a country considered to have an effective but costly healthcare system. For both countries, three domains were reviewed: healthcare system, exercise science graduates' education, and roles played by exercise science graduates in healthcare system. Australia formally recognizes specifically trained exercise science graduates (referred to as Accredited Exercise Physiologists) as healthcare professionals. The exercise interventions they deliver, which were shown to be cost-effective and lead to positive health outcomes, are covered by Medicare, the Australian universal health insurance. However, Medicare covers only a maximum of 5 yearly sessions of all allied-health services taken together. Conversely, Switzerland, despite offering university master's degrees that focus on physical activity delivery to clinical populations, does not recognize the respective graduates as healthcare providers. As a result, their services are not covered by the Swiss health insurances. The latter do, however, cover a generous number of services (not formally limited) delivered by other allied-health professionals. In conclusion, Australia makes a better use of exercise science graduates than Switzerland does. Switzerland would benefit from establishing a clinical profession for exercise science graduates, defining competencies that they should acquire and setting their scope of practice. The very restricted number of therapy sessions covered by Medicare might limit the positive impact exercise science graduates have on the Australian healthcare system. Overall, mutual learning between countries can promote development and global recognition of clinical positions for exercise science graduates.

## Introduction

Physical inactivity (PI) is a leading risk factor for global mortality worldwide and a major preventable cause of non-communicable diseases (NCDs) (Kohl et al., 2012; Lee et al., 2013; Murray et al., 2020; Ramirez Varela et al., 2021). In 2008, PI accounted for more than 5.3 million of the 57 million deaths worldwide (Lee et al., 2012). As a result, PI imposes an important societal and economic burden on healthcare systems (Ding et al., 2016). Fortunately, evidence suggests that prescribing physical activity (PA) is effective to reduce PI, prevent and treat most NCDs (Khan et al., 2012). Accordingly, the World Health Organization (WHO) released in 2018 the “global action plan on physical activity” which aims to reduce global prevalence of PI in adults and adolescents by 15% by the year 2030 (World Health Organization, 2018a).

Patients suffering from or being at risk for NCDs would benefit from effective and safe physical activity interventions delivered by exercise science graduates (Franklin et al., 2009; Pearce and Longhurst, 2021). While the integration of exercise science graduates in healthcare systems has received little attention in the scientific literature so far, Australia is commonly cited as a successful model in this regard (Franklin et al., 2009; Maiorana et al., 2018; Zhou et al., 2019; Pearce and Longhurst, 2021). This topic has not been investigated for Switzerland yet, whose healthcare system is recognized for its effectiveness (albeit costliness) (Ebert et al., 2017). The present work aims to review and compare the role played by exercise science graduates in the Australian and Swiss healthcare systems. In our work, we define an exercise science graduates as a university graduate (bachelor or master degree) trained in the field of sport and exercise science. As there is no unified nomenclature to designate such a profession, we decided to favor the general term “exercise science graduate” over country-specific terms to acknowledge that these professionals undertook scientific training at university in the field of physical exercise. Graduates with degrees from related fields, such as but not limited to physiotherapy, physical education, sports psychology, sports management as well as social and human sciences applied to sport were not considered in the present work.

## The Australian and Swiss Health Care Systems

As shown in Table 1, Australia has more physicians (Australian Institute of Health and Welfare, 2016; Ärztedichte im ambulanten Sektor Swiss Federal Statistical Office, 2019; Federal Statistical Office, 2019b), but fewer hospital beds per 1,000 inhabitants than Switzerland (Krankenhausbetten, 2018; Australian Institute of Health and Welfare, 2019a). While NCDs are more prevalent in Australia, the associated burden of disease is similar in both countries (De Pietro et al., 2015; Australian Institute of Health and Welfare, 2018; Federal Statistical Office and Federal Department of Home Affairs, 2018; World Health Organization, 2018b). The Swiss have a longer life expectancy and are more physically active than the Australians (Australian Institute of Health and Welfare, 2019b,c; Federal Statistical Office, 2019d, 2020a). With 78% of its population fulfilling the WHO's recommendation on PA, Switzerland is one of the most physically active nations in Europe (Lamprecht et al., 2020). This implies, however, that 22% of the Swiss population is still insufficiently physically active (Lamprecht et al., 2020). In 2013, PI was responsible for 2.0% of disability-adjusted life years (DALYs), ca. 2,900 premature deaths, 2.1 million cases of incident illness, and 2.4 billion Swiss francs (ca. 2.6 billion American dollars, USD) of direct treatment costs per year in 2013 (Federal Office of Sport, 2013; Mattli et al., 2019). Conversely, 55% of the Australian population is insufficiently physically active (Australian Institute of Health and Welfare, 2019c), with PI causing 5'200-11'489 deaths per year as well as 1.2–5.5% of DALYs (Crosland et al., 2019).

**Table 1:** The Australian and Swiss healthcare systems by the numbers.

	**Australia**	**Switzerland**
Population (in millions)	25.46 (Australian Bureau of Statistics, 2019b)	8.54 (Federal Statistical Office, 2019a)
GDP per capita (ca. in USD)	49,987 (Australian Bureau of Statistics, 2019a)	84,282 (Federal Statistical Office, n.d)
Health expenditure per capita (ca. in USD)	4,989 (Australian Institute of Health and Welfare, 2019d)	10,166 (Federal Statistical Office, 2019d)
Health care expenditure (% of GDP)	10% (Australian Institute of Health and Welfare, 2019d)	12.3% (Federal Statistical Office, 2019d)
Number of physicians per 1000	3.92 (Australian Institute of Health and Welfare, 2016)	2.26 (Federal Statistical Office, 2019b)
Number of hospital beds per 1000	3.9 (Australian Institute of Health and Welfare, 2019a)	4.4 (Federal Statistical Office, 2019c)
Life expectancy at birth (men)	80.5 years (Australian Institute of Health and Welfare, 2019b)	81.4 years (Federal Statistical Office, 2019d)
Life expectancy at birth (women)	84.6 years (Australian Institute of Health and Welfare, 2019b)	85.4 years (Federal Statistical Office, 2019d)
Life expectancy at age 65 (men)	19.7 years (Australian Institute of Health and Welfare, 2019b)	19.9 years (Federal Statistical Office, 2020b)
Life expectancy at age 65 (women)	22.3 years (Australian Institute of Health and Welfare, 2019b)	22.7 years(Federal Statistical Office, 2020b)
Prevalence of NCD	12.7 million (50%) (Australian Institute of Health and Welfare, 2018)	2.2 million (26%) (Federal Statistical Office and Federal Department of Home Affairs, 2018)
Burden of NCD^a^	89% (World Health Organization, 2018b)	90% (World Health Organization, 2018b)
Prevalence of PI^b^	55% (Australian Institute of Health and Welfare, 2019c)	22% (Lamprecht et al., 2020)
Type of health insurance	Universal public health insurance	Mandatory private health insurance

*USD, American dollars; GDP, gross domestic product; NCD, non-communicable disease; PI, physical inactivity*.

a *Estimation of the percentage of deaths caused by NCDs*.

b *<150 min of moderate to vigorous activity per week*.

Healthcare costs as a proportion of gross domestic product (GDP) are higher in Switzerland than in Australia (Australian Bureau of Statistics, 2019a; Federal Statistical Office, n.d). Indeed, health expenditure amounted to 10% of the Australian GDP in 2017–2018, which corresponds to 185.4 billion Australian Dollars (AUD) (ca. 123.1 billion USD) (Australian Institute of Health and Welfare, 2019d). In Switzerland, healthcare cost corresponded to 12.3% of GDP in 2017, which represents 82.5 billion Swiss francs (ca. 85.6 billion USD). The Swiss healthcare expenditure has risen continuously over the last 15 years (Federal Statistical Office, 2019e), becoming one of the most expensive healthcare systems worldwide. Among countries making up the Organization for Economic Cooperation and Development (OECD), only the United States of America dedicate a greater percentage of its GDP to healthcare expenditure (Federal Statistical Office, 2019d).

### The Australian Health Insurance System

Medicare is Australia's universal health insurance scheme (Australian Department of Health, 2019). It guarantees all Australians, New Zealanders and permanent Australian residents, access to public hospital and other health services, such as consultation with medical doctors, allied-health professionals and access to medication, at low or no cost (Services Australia and Australian Government, 2020a). Medicare is also available to citizens from eleven countries with reciprocal agreements, if they visit Australia and enroll in Medicare (Services Australia and Australian Government, 2019). In addition to Medicare, it is possible to have private health insurance, which reimburses extra cost such as hospitalization in a private setting or some health-related services not reimbursed by Medicare such as dental and optical services (Australian Department of Health, 2019).

The medical services covered by Medicare are listed in the Medicare Benefits Schedule (MBS). While clinicians set their own fees for their services, the MBS determines, for each service, a Schedule fee that corresponds to the amount a service should cost according to the Australian Government. Medicare rebates are then calculated as a percentage of the Schedule fee. For instance, 100% of the Schedule fee for consultations provided by General Practitioner (GPs) are reimbursed, while 85% of the Schedule fee for consultations provided by medical specialists are covered (Medicare Benefits Schedule, 2021a). The difference, if any, has to be paid by the insurance (Australian Medical Association, 2015). Once an insurance out-of-pocket costs rise above a certain threshold determined yearly, Medicare can cover additional cost. The Extended Medicare Safety Net (EMSN) provides families, which are eligible for Family Tax Benefit and concession card holders, with further benefits (Australian Medical Association, 2015).

In addition, the Department of Veteran's Affairs (DVA) provides veterans, war widows/widowers, eligible defense and police force members, and their dependents and caretakers, with a wide range of health services (Health Services for the Veteran Community, 2021). Generally, the DVA covers only services listed on the MBS. Benefits provided by the DVA depend on the category of veteran healthcare cards an individual is holding (Veteran healthcare cards, 2021).

### The Swiss Health Insurance System

In Switzerland, it is compulsory for every resident to have a health insurance (Federal Office of Public Health, 2020a). This also applies to foreigners with a resident permit (Federal Office of Public Health, 2020b). Fifty-eight health insurance companies are accredited by the Federal Government (Federal Office of Public Health, 2020c). The government defines all services that must be covered by these private health insurance companies, which are obliged to accept every eligible applicant. The Swiss health insurance covers most medical services due to illness, accident and maternity (Federal Office of Public Health, 2020a). To complement the mandatory basic health insurance, it is possible to contract a supplementary health insurance, which covers additional services such as but not limited to dental care or access to private hospitals (The Swiss Authorities Online and Federal Chancellery, 2013).

For an insurant, the mandatory health insurance costs are a combination of a premium, a deductible and a retention fee (Federal Office of Public Health, 2018). All the medical services must be paid for out-of-pocket by the insurance up to the deductible. As soon as the deductible is reached, the insurance must pay a retention fee of ten percent on any additional medical services. If the yearly costs of the retention fee exceed a certain amount, the insurance will cover the remaining costs. The premiums depend on the deductible, with higher deductible leading to lower premiums and vice versa (Federal Office of Public Health, 2020d). Insurance can choose the deductible freely within a given range decided by the government. Finally, insurance in less fortunate economic situations can benefit from premium reductions (Federal Office of Public Health, 2020e).

## Exercise Science Graduates' Education in Australia and Switzerland

As shown in Table 2, 14 and 24 Australian universities offer bachelor (B.Sc.) and master (M.Sc.) degrees in exercise science, respectively. In Switzerland, seven universities offer BSc degree, while six offer M.Sc. degrees in exercise science. In both countries, exercise science graduates can enroll in advanced study programs, which include Master of Advanced Studies (MAS), Certificates of Advanced Studies (CAS), Diploma of Advanced Studies (DAS), and Doctor of Philosophy (PhD). The major distinction between both countries lies in the existence of Exercise & Sport Science Australia (ESSA), which is the national body representing Australian exercise science graduates (Exercise and Sport Science Australia, n.d). ESSA defines professional skills that exercise science graduates need to acquire before beginning to practice in the profession (Exercise and Sport Science Australia, 2021). A comparable body is currently absent in Switzerland.

**Table 2:** Educational and professional facts around exercise science in Australia and Switzerland.

	**Australia**	**Switzerland**
**Educational facts**	
B.Sc. in exercise science (*n*)	33^a^ (Exercise and Sport Science Australia, 2021)	7 (Schlesinger et al., 2015)
M.Sc. in exercise science (*n*)	14^a^ (Exercise and Sport Science Australia, 2021)	6 (Schlesinger et al., 2015)
Advanced study (MAS, CAS, DAS, Ph.D.)	Yes	Yes
**Professional facts**	
Accredited allied-health professions	Yes	No
Coverage by health insurances	Yes	No
Exercise science graduates working in healthcare (%)	58 (Stevens et al., 2018)	4.3 (Schlesinger et al., 2015)
Professional organization	ESSA	SGS/4S SVGS ASP-APA

*B.Sc., Bachelor of Science; M.Sc., Master of Science; MAS, Master of Advanced Studies; CAS, Certificate of Advanced Studies; DAS, Diploma of Advanced Studies; Ph.D., Doctor of Philosophy; ESSA, Exercise and Sport Science Australia; SGS, Sportwissenschaftliche Gesellschaft der Schweiz (German for Swiss Sport Science Association), 4S, Société Suisse des Sciences du Sport (French for Swiss Sport Science Association); SVGS, Schweizerische Verband für Gesundheitssport und Sporttherapie (German for Swiss Association of health-centered sport and sport therapy); ASP-APA, Association Suisse des professionnels en activités physiques adaptées (French for Swiss Association of health-centered sport and sport therapy)*.

a *Courses with full or provisional accreditation by ESSA*.

### Exercise Science Graduates' Education in Australia

ESSA is committed to establishing and promoting career paths for exercise science graduates (Exercise and Sport Science Australia, 2019a). In this way, ESSA has established an accreditation system for exercise science graduates, listing knowledge, skills, attitudes, and values to fulfill in order to obtain these accreditations (Exercise and Sport Science Australia, 2021). ESSA also accredits university program across Australia, which provide exercise science training (Education Providers, 2017). ESSA distinguishes between four accredited professions: Accredited Exercise Physiologist (AEP), Accredited Exercise Scientist (AES), Accredited Sport Scientist (ASS), and Accredited High Performance Manager (AHPM) (Exercise and Sport Science Australia, 2019a).

AEPs are university-trained exercise professionals with either a 4-year B.Sc. degree or a 3-year B.Sc. degree plus a 1-year graduate diploma or 2-year M.Sc. degree (Exercise and Sport Science Australia, 2015). These degrees usually include 140 h of practical experience focused on fitness improvement and prevention of chronic conditions and 360 h of practical experience with patients living with chronic conditions should (Exercise and Sport Science Australia, 2015). If these placements are not embedded in the above-mentioned degrees, students need to complete them separately in order to earn the AEP accreditation (Exercise and Sport Science Australia, 2015).

AESs are graduates with a 3-year B.Sc. degree. Unlike AEPs, AESs need to complete the 140 h of practical experience focused on fitness improvement and disease prevention but not the 360 h of clinical practical experience^1^. Indeed, AESs mainly deliver preventive exercise programs to healthy populations (see text footnote 1). To deliver services to people with existing pathologies, AESs need a prescription issued by an AEP, a physiotherapist or a physician^2^ (see text footnote 1) AESs are trained to recognize the necessity to refer a patient to another related professional such as a physiotherapist, an AEP or a physician (Exercise and Sport Science Australia, 2017). Finally, ASSs deliver exercise interventions to recreational and elite athletes, while AHPMs operate mostly in the field of high-performance sport (Exercise and Sport Science Australia, 2019b).

### Exercise Science Graduates' Education in Switzerland

Switzerland Misses an Equivalent to ESSA. The Swiss Sport Science Association [in German: *die Sportwissenschaftliche Gesellschaft der Schweiz* (SGS), in French: *Société Suisse des Sciences du Sport* (4S)] is the scientific body organizing Swiss sport and exercise science graduates, but it neither sets the skills and knowledge future exercise science graduates should acquire nor defines professions or delivers accreditations for exercise science graduate (Sportwissenschaftliche Gesellschaft der Schweiz, 2020). Two regional bodies, namely the Swiss Association of health-centered sport and sport therapy (in German: *Schweizerische Verband für Gesundheitssport und Sporttherapie*), in the German-speaking part of Switzerland, and the Swiss Association of professionals in adapted physical activity (in French *Association Suisse des professionnels en activités physiques adaptées*), in the French-speaking part of Switzerland, are currently working together toward a common definition of a profession dedicated to graduates trained in the field of sport and exercise science. However, results of this work have not been published yet. As a result, the term *exercise science graduate* (or equivalent) is not protected.

B.Sc. degrees in exercise and sport science typically take 3 years to complete as a full-time student. While their curricula are designed to open access to a wide spectrum of professional areas spanning from sports economy to exercise therapy, the focus is clearly put on training of future physical education schoolteachers. The university of Basel also offer a B.Sc. degree focusing on physical activity delivery to clinical populations^3^. Students with interest in exercise science can subsequently enroll in a 2-year M.Sc. degree. Specifically, M.Sc. degrees focusing on prevention and health promotion and adapted physical activity are offered^4^,^5^,^6^,^7^ (Universität Bern, 2019; Federal Office of Sport, n.d). These graduates are trained to clinically assess and counsel patients about active and healthy lifestyle (see text footnotes 6,7) (Universität Bern, 2019, 2020a). Additionally, they acquire the skills to program and deliver exercise interventions for specific clinical populations (Universität Bern, 2020b). However, uniform requirements across MSc degrees in exercise science are lacking and practical education is insufficient in comparison to training of other health professionals. For trainees, it means that they are leaving university without having been educated for a concrete profession. Consequently, foundations for a potential federal accreditation as “health professionals” are currently missing.

## Exercise Science Graduates in the Australian Healthcare System

In Australia, 58% of Exercise Science graduates work in the health sector (Stevens et al., 2018). AESs mainly work in a preventive setting, while AEPs can deliver exercise intervention both to healthy individuals (prevention) and patients (management and treatment). Under particular circumstances (i.e., a prescription has been issued by an AEP, a physiotherapist or a physician), AESs are allowed to deliver exercise therapy to patients (see text footnote 1).

AEPs have foundational knowledge to prevent, treat, and manage health conditions (including diseases, disorders, traumas and injuries), including chronic conditions (Exercise and Sport Science Australia, 2015). Indeed, they are trained to interpret referral information, perform clinical screenings, assess patients' functional capacity, and use behavioral change skills to encourage patients opting for a healthy and physically active lifestyle (Exercise and Sport Science Australia, 2015). The AEP's scope of practice also encompasses the design and implementation of effective exercise interventions within groups of patients (Exercise and Sport Science Australia, 2018). However, they are not authorized to make clinical diagnostics, prescribe medicines, perform joint manipulation, massages, and ultrasound therapy (Smart et al., 2016). AESs are skilled to screen and assess fitness and performance capacity, to design and deliver exercise-based interventions to prevent injury, manage risk factors for chronic conditions, improve fitness, and performance. They also provide physical activity education and general nutritional advice (see text footnote 1).

Unlike AESs, AEPs are officially recognized as allied-health professionals and therefore are authorized to deliver Medicare-rebated exercise therapy to patients living with chronic diseases (Exercise and Sport Science Australia, 2019b,c). Importantly, Medicare covers only a maximum of five therapy sessions per year and this includes not only exercise therapy but also other medical services such as physiotherapy, osteopathy, podiatry, and chiropractic services (Smart et al., 2016). Due to this limitation, the average AEP provided <3 Medicare-covered consultations in 2016 (Smart et al., 2016).

For exercise physiology services provided by AEPs (MBS item 10953), the Scheduled fee is 64.80 AUD (ca. 47 USD) and the Medicare benefit is 85% (55.10 AUD, ca. 40 USD) (Commonwealth of Australia, Australian Department of Health, 2019a). The duration of this service should be of at least 20 min (Commonwealth of Australia, Australian Department of Health, 2019a). In addition, Medicare also covers up to eight exercise therapy sessions for patient with type 2 diabetes mellitus (T2DM, MBS item 81115) (Commonwealth of Australia, Australian Department of Health, 2019b). This service is delivered to groups of 2 to 12 persons. The Scheduled fee corresponds to 20.70 AUD (ca. 15 USD) and the Medicare benefit is 85% (17.60 AUD, ca. 13 USD) (Commonwealth of Australia, Australian Department of Health, 2019b). The service should last at least 60 min (Commonwealth of Australia, Australian Department of Health, 2019b). Before enrolling in such an exercise therapy, a patient needs to be assessed by an AEP, during which patient's risk, exercise and functional capacity in relation to chronic disorders are evaluated (MBS item 81110) (Exercise and Sport Science Australia, 2015). These assessments are covered once per calendar year, the Scheduled fee corresponds to 83.10 AUD (ca. 60 USD) and the benefit is 85% (70.65 AUD, ca. 51 USD) (Commonwealth of Australia, Australian Department of Health, 2019c). The assessment should last at least 45 min (Commonwealth of Australia, Australian Department of Health, 2019c). Additional MBS items are available for exercise physiology services delivered by AED to residents of an aged care facility (items 93504, 93518, 93527, 93607, 93614, and 93620) and to persons who are of Aboriginal or Torres Strait Islander descent (items 81315, 93549, 93571, and 93582) (Exercise physiology in the MBS, 2021). Importantly, referral by a physician is required for the Medicare benefit. Lastly, it should be acknowledged that the DVA also covers service delivered by AEPs with benefits depending on the category of veteran healthcare cards a patient is possessing (Exercise Physiology services, 2021).

ESSA mandated Deloitte Access Economics to estimate the benefits of employing AEPs to manage T2DM and pre-diabetes, major depressive disorder, and cardiovascular diseases (CVD) (Deloitte, 2016). Strikingly, it was demonstrated that the benefit-cost ratio (BCR) with reference to direct health care expenditure and the average cost of exercise interventions, was 8.8 to 1 per patient with T2DM, 6.2 to 1 per patient with CVD, 6.0 to 1 per patient with pre-diabetes, and 2.7 to 1 per patient with depression (Deloitte, 2016). As a reflection of these impressive figures, number of exercise therapy services delivered by AEPs under MBS coverage since 2006 have been steadily increasing (Services Australia and Australian Government, 2020b). Yet, <1% of Australians living with T2DM are referred to an AEPs, according to a governmental report (Australian Institute of Health and Welfare, 2019e). Likewise, according to the limited literature available, AEPs seem to be only marginally involved in cardiac and pulmonary rehabilitation programs nationwide (Johnston et al., 2011; Jackson et al., 2018). A possible explanation for the low referral rate might be, that medical doctors are not aware of AEPs' knowledge and skills and/or of the scientific evidence around physical activity to prevent and treat NCDs (Smart et al., 2016; Craike et al., 2019). While referrals rate to AEPs remain low, a study among 6827 Australian GPs revealed an increased in referrals from 0.38 per 1,000 encounters in 2009 to 1.44 per 1,000 encounters in 2016 (Craike et al., 2019). Interestingly, half of the referrals were made through Chronic Disease Management (CDM) plans, which enable GPs to plan and coordinate multidisciplinary patients' care (Craike et al., 2019). The authors suggested that the fee claimable by GPs when using CDM plans could be an effective incentive explaining partly the observed increased referrals (MBS item 723, Scheduled fee 118.95 AUD or ca. 86 USD) (Craike et al., 2019; Medicare Benefits Schedule, 2021b).

## Exercise Science Graduates in the Swiss Healthcare System

Exercise science graduates are not recognized as healthcare professionals according to the Swiss legislation (Bundesgesetz, 2020). Neither are exercise science curricula mentioned in the federal regulation on the accreditation of university medical curricula (Verordnung, 2007). As a consequence, exercise science graduates are not mentioned among the professionals a patient can be referred to Federal Office of Public Health (2020f), leading to only 4.3% of all exercise science graduates working in the health sector (Schlesinger et al., 2015). These exercise science graduates are involved either in isolated clinical pilot projects or in disease-specific rehabilitation programs, as illustrated hereafter. Additionally, rehabilitation and sport clinics also employ a very limited number of exercise science graduates specialized in adapted physical activity or prevention and health promotion. As health insurers usually do not cover their services, coverage is handled on a case-by-case basis.

An interesting pilot program (named *pas à pas*, French for step by step) is ongoing in one of the 26 Swiss cantons (*Canton de Vaud*). Physicians can refer patients suffering from NCDs to exercise science graduates owning a master degree in adapted physical activity^8^. During this three- to six-month exercise therapy, exercise science graduates evaluate the patient's fitness and needs, elaborate a personalized exercise program, and inform the referring physician about the patient's progress. Funding of this pilot project, provided by the federal-funded foundation *Health Promotion Switzerland*, is time-limited (see text footnote 8). Patients involved in the project reported increased well-being and physical activity levels (Lociciro and Bize, 2017).

DIAfit (contraction of diabetes and fitness) is a 12-week exercise program targeting patients with diabetes and pre-diabetes^9^ The program encompasses education regarding active and healthy living, diabetes counseling, and nutritional advice. Three weekly sessions of exercise therapy are also included and can be delivered by physiotherapists, PE teachers and exercise science graduates as long as they followed a 6-day postgraduate course^10^. There are no data on the proportion of exercise science graduates involved in DIAfit. The program's costs are covered by health insurance companies.

Exercise science graduates can also deliver exercise therapy within cardiovascular rehabilitation program, if they complete a 300-h long certificate of advanced studies in cardiac rehabilitation^11^. As for DIAfit, no data is available on the number of exercise science graduates involved (Schweizerische Herzstiftung, n.d). After a first phase of light mobilization taking place in hospital, patients enter a 12-week ambulatory program made of two to three weekly training sessions (Saner, 2012). Alternatively, patients can spend three to four weeks in a specialized rehabilitation clinic, where exercise interventions are delivered on a daily basis (Saner, 2012). Finally, patients have the possibility join so-called heart groups, which usually organize one exercise session per week. Health insurances cover the costs of the first two phases only (Saner, 2012).

Even though widely recommended, exercise intervention programs for cancer patients are generally not covered by basic health insurance (Hayes et al., 2019). The Swiss Cancer League offers an exercise therapy program delivered by physiotherapists or exercise science graduates after completion of a specific CAS/DAS (Schweiz, 2021). Local initiatives, such as PASTEC (French acronym for Promotion of Therapeutic Sports Activity for Children with Cancer) also employs exercise science graduates to deliver physical activity intervention to children with cancer (Cornevin and Carrard, 2017). PASTEC is pilot program mainly financially supported by diverse private associations.

## Discussion

**Figure 1:**
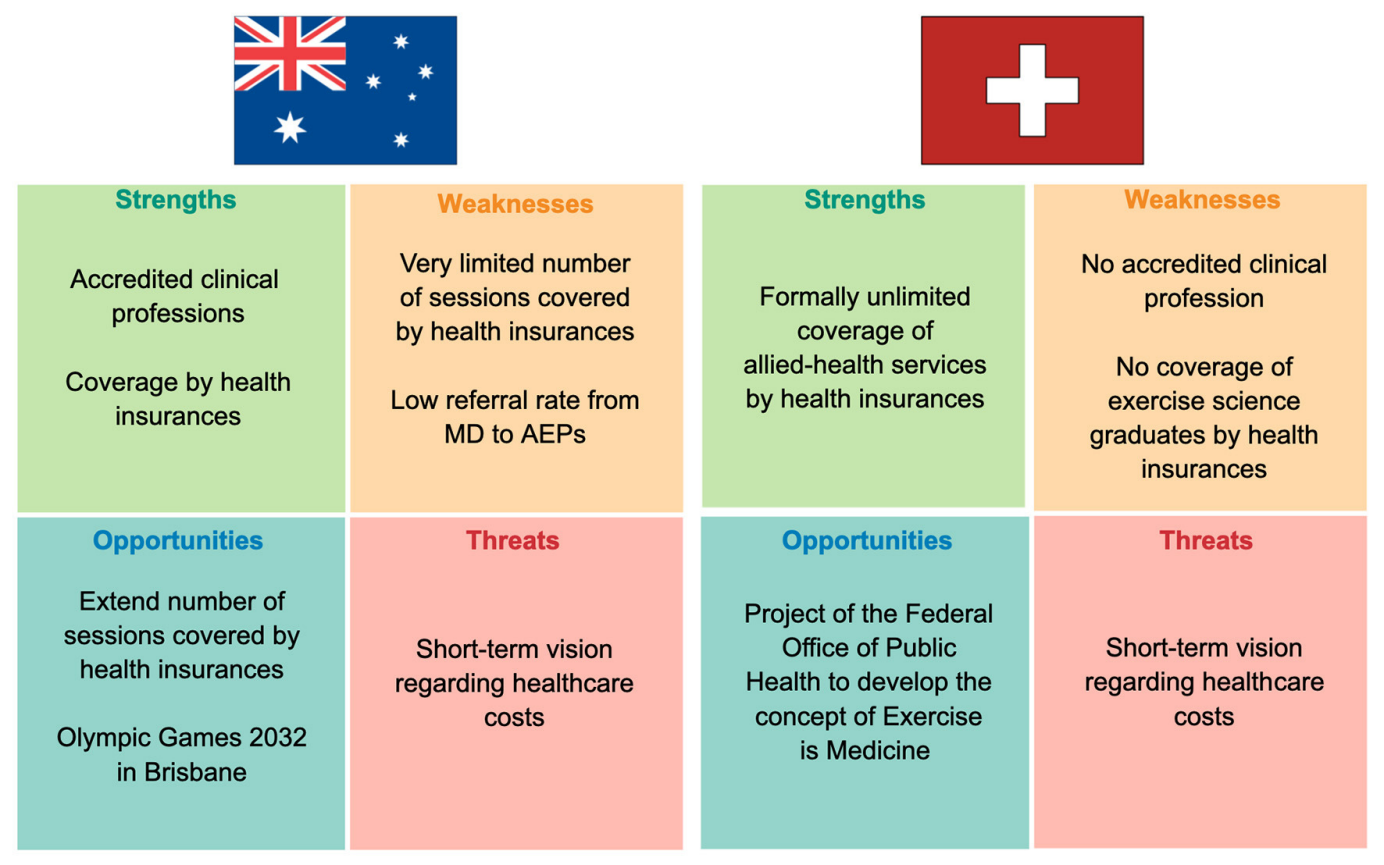
Exercise science graduates in the Australian and Swiss healthcare systems: strengths, weaknesses, opportunities and threats analysis. MD, medical doctor; AEP, accredited exercise physiologist.

As illustrated in the summarizing Figure 1, Australia defines accredited clinical professions for exercise science graduates, whose skills and scope of practice are clearly and precisely defined by ESSA (Exercise and Sport Science Australia, 2018). AEPs are officially recognized as health professionals, which enables them to deliver services covered by Medicare to patients living with NCDs (Deloitte, 2016). Strikingly, it was demonstrated that employing AEPs to prevent and manage NCDs is both medically efficient and cost-effective (Deloitte, 2016). The principal identified weakness of the Australian system lies in the very limited number of allied-services covered yearly by Medicare (Commonwealth of Australia, Australian Department of Health, 2019a). The latter implies a competitive reimbursement scheme between allied-health professionals and might limit patients' access to AEPs (Smart et al., 2016). Low referral rate from medical doctors to AEPs represents another identified weakness (Smart et al., 2016). Both issues challenge many AEPs to find other sources of income or provide exercise services without MBS rebate (Smart et al., 2016). Educating current and future medical doctors about the potency of physical activity to prevent and treat NCDs would certainly help to improve the referral rate. Currently, Australian medical students received little education in sport and exercise medicine (Strong et al., 2017). Indeed, if most Australian medical schools plan to educate their students about PA, a mere 1.7 h of PA education is delivered per year on average (Strong et al., 2017). Furthermore, important aspects such as strength training are mostly ignored (Strong et al., 2017). Regarding GPs, it has been reported that their exercise recommendations lack of specificity and that recommendations are mainly done in a secondary rather than in a primary prevention setting (Short et al., 2016). The recent attribution of the 2032 Summer Olympic Games to Brisbane might represent a unique opportunity for Australia to strengthen AEPs' roles and use Olympic legacy to elevate physical activity on a population level (Bauman et al., 2021).

Unlike Australia, Switzerland lacks a defined and recognized clinical profession for exercise science graduates (Figure 1). As a consequence, exercise science graduates are only marginally involved in the Swiss healthcare system and their services are generally not covered by health insurances (Schlesinger et al., 2015). However, pilot program, such as *pas à pas*, shows that integrating exercise science graduates in the Swiss healthcare system will improve it for the best of patients' health and likely reduce long-term health-related costs (Lociciro and Bize, 2017). A recent report elaborated by health experts on the mandate of the Swiss Federal Office of Public Health concluded that physical activity as a prevention and treatment strategy is currently underused in Switzerland (Nast et al., 2020). Recommendations were made to create an umbrella organization called “Exercise is Medicine Switzerland,” which should set the basis for prevention and therapeutical exercise prescription (Nast et al., 2020). In this context, we firmly believe that Switzerland will benefit from creating a clinical profession for exercise science graduates. Importantly, exercise science graduates could help the 22% physically insufficiently active Swiss to fulfill PA recommendation. Common reported barriers in this group of population include a lack of time, work overload and health issues (Lamprecht et al., 2020). These are problems, exercise science graduates are trained to deal with, for instance by tailoring exercise program to patient's health condition or by emphasizing the utility of interval training in case of a lack of time (Maiorana et al., 2018).

While this study focused on two countries, the integration of exercise science graduates into healthcare is of global interest. Indeed, articles advocating for a better recognition of this profession have been recently published by colleagues from China (Zhou et al., 2019), the United Kingdom (Jones et al., 2021) and the USA (Berry et al., 2021). Moreover, societies for clinical exercise physiology have been established in several countries, including the UK^12^, the USA^13^, Canada^14^, and New Zealand^15^. France established a national society for “adapted physical activity”^16^, while South Africa has a society for “biokinetics”^17^. Specialist in “adapted physical activity” and “biokinetics” are respectively the French and South African equivalent to the term clinical exercise physiologist used in the above-mentioned countries. A common issue is that implementing and developing the concept of “Exercise is Medicine” in healthcare systems requires financial and educational investment before achieving long-term reduction of health-related costs (Deloitte, 2016; Ding et al., 2016). Thus, a short-term healthcare vision represents a constant threat, which should be combatted by all stakeholders involved in sport and exercise medicine. These healthcare professionals as well as their respective colleges, associations and societies should collectively and collaboratively advocate for the implementation of the *Exercise is Medicine* concept into clinical practice. Furthermore, they should join their forces to support the creation and further development of clinical professions and positions for university graduates with degree in exercise and sport science. It is also necessary to deepen undergraduate education on sport and exercise medicine at medical, nursing, physiotherapy, and other allied-health schools (Carrard et al., 2019).

## Conclusion

PA is a cornerstone in the prevention, treatment, and rehabilitation of multiple NCDs. Exercise interventions delivered by exercise science graduates have been shown to lead to positive health and socioeconomic outcomes. Australia officially recognizes specifically trained exercise science graduates as healthcare providers. Consequently, the services they deliver are covered by Medicare, the Australian universal health insurance. While the integration of exercise science graduates in the Australian healthcare system is exemplary, educational efforts in medical schools and postgraduate medical training are still necessary to facilitate patients' referral from medical doctors to AEPs. Simultaneously, extending the number of yearly AEP-delivered sessions covered by Medicare is essential to make these services accessible to all patients in need but also to fully exploit AEPs potential. Conversely, Switzerland, despite offering university MSc degrees that focus on PA delivery to clinical populations does not formally recognize the respective graduates as healthcare providers. As a result, they are still poorly integrated in the Swiss healthcare system and their services are not covered by health insurances. Switzerland would benefit from establishing a clinical profession for exercise science graduates, defining the skills and knowledge that they should acquire and setting their scope of practice. These steps appear to be essential to exploit the full potential of clinical exercise science graduates. Finally, mutual learning between countries can facilitate global recognition and development of clinical positions for exercise science graduates.

## Author Contributions

JC and MG: conceptualization, data collection, and formal analysis. JC, MG, and TH: methodology. JC, MG, IC, SM, BG, TH, and AS-T: interpretation of data. JC, MG, IC, and SM: writing—original draft preparation. JC, MG, IC, SM, TH, BG, and AS-T: writing—review and editing. JC and BG: visualization. JC, AS-T, and TH: supervision. JC: project administration. All authors have read and agreed to the published version of the manuscript.

## Conflict of Interest

The authors declare that the research was conducted in the absence of any commercial or financial relationships that could be construed as a potential conflict of interest.

## Publisher's Note

All claims expressed in this article are solely those of the authors and do not necessarily represent those of their affiliated organizations, or those of the publisher, the editors and the reviewers. Any product that may be evaluated in this article, or claim that may be made by its manufacturer, is not guaranteed or endorsed by the publisher.

## Footnotes

^1^Exercise and Sport Science Australia. *Accredited Exercise Scientist Scope of Practice*.

^2^Exercise is Medicine Australia. About us. n.d. Available online at: http://exerciseismedicine.com.au/about-us/.

^3^Studieren am Departement für Sport. *Bewegung und Gesundheit der Universität Basel*. Available online at: https://dsbg.unibas.ch/de/studium/.

^4^Université de Lausanne, Faculté des sciences sociales et politiques (2020). *Maîtrise universitaire ès sciences en sciences du mouvement et du sport*. Available online at: https://www.unil.ch/formations/en/home/menuinst/masters/sciences-du-mouvement-et-du.html.

^5^Universität Bern. Master Sportwissenschaftliche Forschung. Available online at: https://www.philhum.unibe.ch/studium/studienprogramme/master_sportwissenschaftliche_forschung/index_ger.html.

^6^Federal Office of Sport. *Master of Science in Sports Sciences*. [Available from: https://www.ehsm.admin.ch/en/degree-and-further-education/msc-sportwissenschaften.html.

^7^Universität Basel. *Sport, Bewegung and Gesundheit: Studienrichtung Prävention und Gesundheitsförderung Master*. Universität Basel Available online at: https://www.unibas.ch/de/Studium/Studienangebot/Studiengaenge-faecher.html?study=Sport-Bewegung-Gesundheit-Praevention-und-Rehabilitation-MSc.

^8^Pasà, Pas+. Pasà Pas+. Available online at: https://www.pas-a-pas.ch/.

^9^DIAfit. *Rehabilitationsprogramm*. Available online at: http://www.diafit.ch/de/30_rehaprogramm/00_rehabilitation.htm.

^10^DIAfit. Für Therapeuten. Available online at: http://www.diafit.ch/de/54_ausbildung/20_therapeuten.htm.

^11^Swiss Working Group for Cardiovascular Prevention Rehabilitation and Sports Cardiology. Qualitätsanforderungen für die kardiovaskuläre Prävention und Rehabilitation der SCPRS (2018).

^12^Clinical Exercise Physiology UK. BASES. Available online at*:* https://www.bases.org.uk/article-bases_and_cep_uk_announce_clinical_exercise_physiologists_now_eligible.html.

^13^Clinical Exercise Physiology Association. Available online at: https://www.acsm-cepa.org.

^14^Canadian Society for Exercise Physiology. Available online at: https://csep.ca.

^15^Clinical Exercise Physiology New Zealand. Available online at: https://www.cepnz.org.nz.

^16^Société, Française. *des Professionnels en Activité Physique Adaptée*. Available online at: https://www.sfp-apa.fr.

^17^Biokinetics Association of South Africa. Available online at: https://biokineticssa.org.za.
